# The Effects of Dimensions on the Deformation Sensing Performance of Ionic Polymer-Metal Composites

**DOI:** 10.3390/s19092104

**Published:** 2019-05-07

**Authors:** Jiale Wang, Yanjie Wang, Zicai Zhu, Jiahui Wang, Qingsong He, Minzhou Luo

**Affiliations:** 1 School of Mechanical and Electrical Engineering, Hohai University, Changzhou Campus, Changzhou 213022, China; wangjiale63@163.com (J.W.); wangjh0913@163.com (J.W.); 2 Jiangsu Key Laboratory of Special Robot Technology, Hohai University, Changzhou 213022, China; 20161956@hhu.edu.cn; 3 School of Mechanical Engineering, Xi’an Jiaotong University, Xi’an 710049, China; 4 Jiangsu Provincial Key Laboratory of Bionic Functional Materials, Nanjing University of Aeronautics & Astronautics, Nanjing 210016, China; heqingsong@nuaa.edu.cn

**Keywords:** IPMC, sensor, dimension, deformation

## Abstract

As an excellent transducer, ionic polymer-metal composites (IPMCs) can act as both an actuator and a sensor. During its sensing process, many factors, such as the water content, the cation type, the surface electrode, and the dimensions of the IPMC sample, have a considerable impact on the IPMC sensing performance. In this paper, the effect of dimensions focused on the Pd-Au typed IPMC samples with various thicknesses, widths, and lengths that were fabricated and their deformation sensing performances were tested and estimated using a self-made electromechanical sensing platform. In our experiments, we employed a two-sensing mode (both current and voltage) to record the signals generated by the IPMC bending. By comparison, it was found that the response trend was closer to the applied deformation curve using the voltage-sensing mode. The following conclusions were obtained. As the thickness increased, IPMC exhibited a better deformation-sensing performance. The thickness of the sample changed from 50 μm to 500 μm and corresponded to a voltage response signal from 0.3 to 1.6 mV. On the contrary, as the length increased, the sensing performance of IPMC decreased when subjected to equal bending. The width displayed a weaker effect on the sensing response. In order to obtain a stronger sensing response, a thickness increase, together with a length reduction, of the IPMC sample is a feasible way. Also, a simplified static model was proposed to successfully explain the sensing properties of IPMC with various sizes.

## 1. Introduction

As one kind of representative ionic electroactive polymer (iEAP), ionic polymer-metal composites (IPMCs) are known as actuators with light weight, noiseless operation, and larger displacements under low-driving voltage [1,2,3]. Not only can it produce large deformations at low voltage, but IPMC will also generate a corresponding electrical response when subjected to mechanical bending [4,5,6,7]. These characteristics of IPMC have enormous application potential in the fields of biomimetic machine biomedicine, flexible sensing, and micro-electro-mechanical system (MEMS) [8,9,10,11,12].

**Figure 1:**
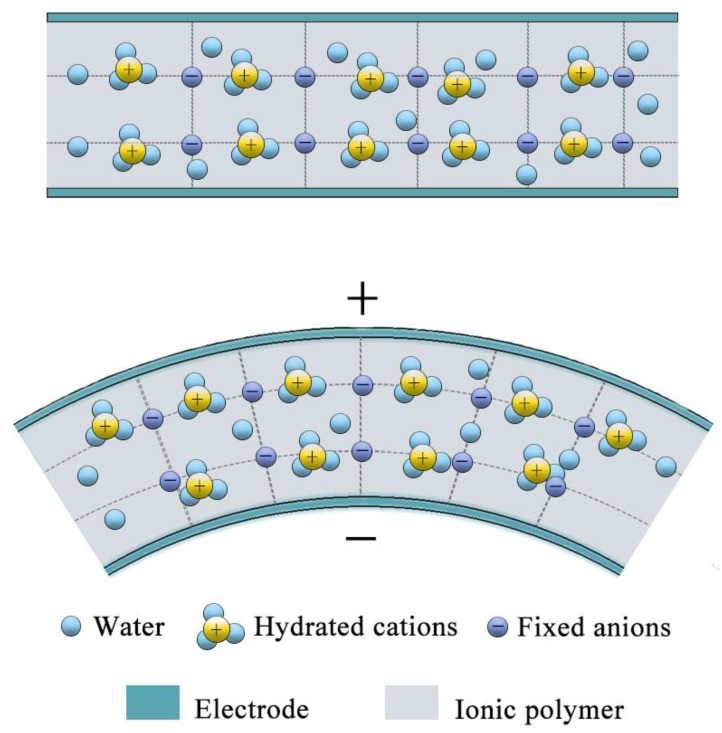
Ionic polymer-metal composite (IPMC) sensing under a bending deformation.

As shown in Figure 1, a typical IPMC has a sandwich structure, composed of a ionomer membrane and two metallic electrode layers [13]. The polymer membrane contains mobile hydrated cations, water as solvents, and fixed anions. Generally, when an IPMC is subjected to mechanical bending, the hydrated cations move towards the expanded region [14,15]. This means that the potential of the electrode layer on the expanded side will be higher than the other side, as shown in Figure 1.

In generally, IPMC is capable of sensing and operating in harsh conditions. IPMC materials have a lot of excellent properties, such as insensitivity to magnetic fields and simple fabrication processes, one order of magnitude more sensitive than traditional piezoelectric transducers. IPMC still has drawbacks, and the voltage generated by IPMC (0.01–10 mV) is still much lower than polyvinylidene fluoride (PVDF) film, which is a typical flexible sensor [16].

As early as the 1990s, the sensing abilities of IPMC have been reported to gradually cause widespread concern, and many researchers have conducted in-depth research on this. Shahinpoor et al. [17] confirmed the linear relationship between bending displacement and output voltage of strip-typed IPMC [18]. Konyo et al. investigated the voltage-response dependence of the frequency and revealed that a velocity of applied bending was in proportion to the output voltage of IPMC [19]. Takagi et al. showed that the voltage response of IPMC increased with frequency in the low-frequency range [20]. Regarding the influence of the ambient humidity on IPMC, Zhu et al. proposed a comprehensive physical interpretation of the dependence of water content [21]. By changing the composition of IPMC, Liu et al. tried to replace the electrode and ionic solvent inside IPMC with graphene and ionic liquid, respectively, and obtained an output electrical signal of 4.5 millivolts [22]. In fact, in addition to the water content, the bending displacement, the velocity of deformation, the type of cation, and the surface electrode, the dimensions have a considerable influence on the sensing performance of IPMC. However, due to the complexity of IPMC structure, it is difficult to fully explain its sensing mechanism and further build an explicit physical model. 

In this paper, we employed impregnation-reduction plating (IRP), together with electroplating (EP), to fabricate IPMC samples with various dimensions, and then measured their sensing performances under dynamic bending load. Our paper was organized as follows: firstly, we fabricated IPMC samples with specific sized series, and then compared this to the voltage response and the current response to evaluate the deformation sensing performance of IPMC by voltage and current amplifier, respectively. Finally, the effects of the dimensions on IPMC were investigated, including thickness, length, and width. The increase in the thickness and length of IPMC had obvious improvements on the sensing performance, while the width had little effect on the sensing signal. 

## 2. Experimental Methods

### 2.1. Fabrication of IPMC

**Figure 2:**
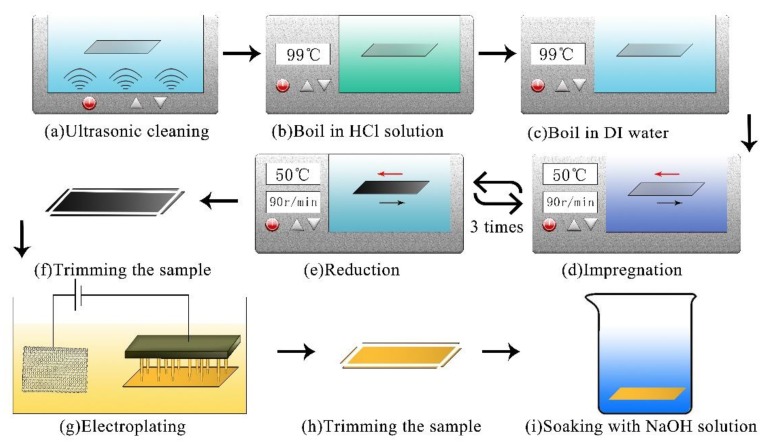
The detailed fabrication process of IPMC.

In this experiment, ionomers (Nafion from Dupont, DE, USA and GEFC from golden energy fuel cell (GEFC) corporation, Beijing, China) were used as the interlayer of IPMC, which had the same microstructure as that generated by the copolymerization of a perfluorinated vinyl ether comonomer with tetrafluoroethylene (TFE). As shown in Figure 2, the preparation process developed by our lab was as follows [23].

Pre-treatment step: after being ultrasonic cleaning in deionized (DI) water (30 min, 60 °C), as shown in Figure 2a, the membrane was successively immersed in 2 mol/L HCL (99 °C) and DI water (99 °C) for 30 min (Figure 2b,c). The aim of this process was to purify the base ionomer membrane.

Impregnation-reduction plating (IRP) step: as shown in Figure 2d, the pretreated membrane was placed in an ammonia solution of Pd(NH_3_)_4_Cl_2_ (0.01 mol/L, 50 °C) for 60 minutes within a water bath thermostat oscillator (50 °C, 80–90 r/min). After being rinsed in deionized (DI) water, the membrane was immersed in NaBH_4_ solution (0.02 mol/L, pH > 13) for 30 min with a stirring speed of 80–90 r/min. The membrane was rinsed once again and repeated the IR step twice. This process was done to incorporate the palladium complex cations into the polymer, which were reduced into the palladium particles in or near the inner surface of the base membrane (Figure 2e).

Electroplating (EP) step: we built an electroplating set-up, which consisted of a titanium anode, DC power supply, and an array of cathodes with conductive spring pins, as shown in Figure 2g. To prevent short circuiting on both sides of the IPMC, the sample needed to be trimmed before the electroplating (Figure 2f). The source of Au^+^ was provided by a gold electroplating solution with the mass concentration of 1.2 g/L. In order to ensure the uniformity of the surface resistance, the electroplating was carried out under 5 V at a current intensity of 0.05–0.15 A for 20–30 s on each side, and repeated 5–10 times. In order to avoid the effect of the surface resistance of the IPMC on the sensing response, the electroplating time of each sample was controlled to keep the surface resistances of the IPMC equal. This process was used to further decrease the surface resistivity.

Post-processing step: after the IR step, the sample was trimmed again and then the samples were immersed in an NaOH solution (0.2 mol/L) within a water bath thermostat oscillator (50 °C, 80–90 r/min) for 120 min. Finally, they were preserved in deionized (DI) water.

**Figure 3:**
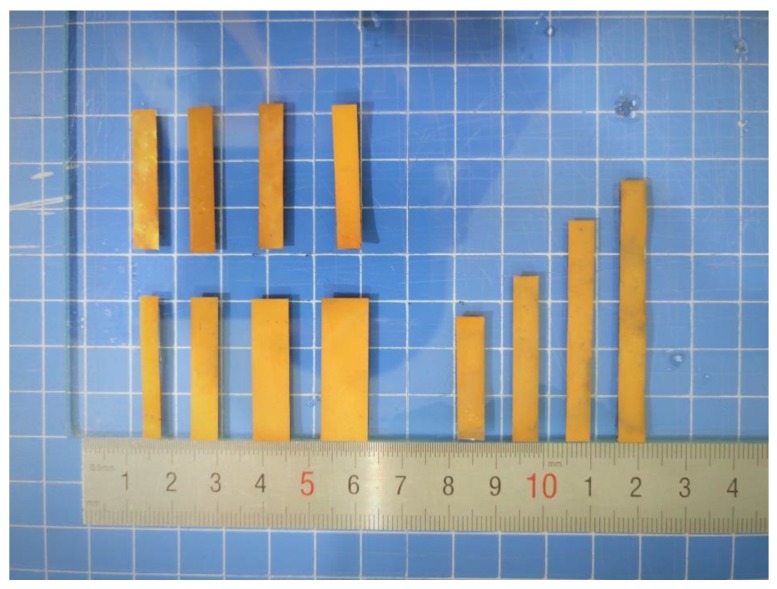
IPMC samples with dimension difference.

**Figure 4:**
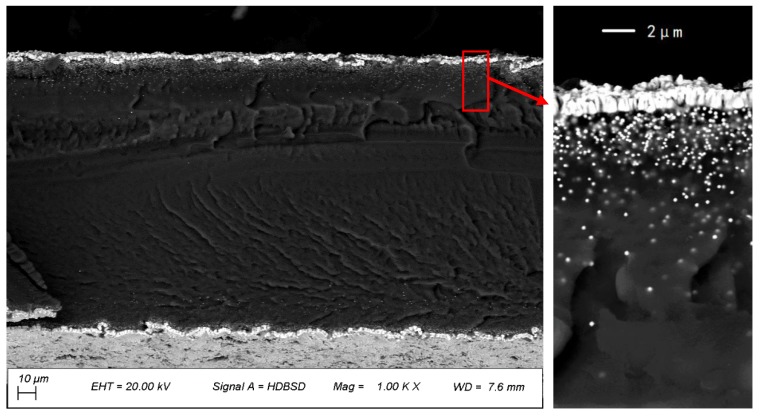
SEM cross-section of the samples (L2).

After the preparation steps were finished, the IPMC was cut into the needed sizes and numbered, as shown in Figure 3 and Table 1 (T, L, W represent thickness, length and width, respectively). The cross-section of the samples were observed by scanning electron microscopy (SEM, sigma 500), and the SEM cross-section of the sample (L2) is shown in Figure 4.

### 2.2. Electromechanical Sensing Test Platform

**Figure 5:**
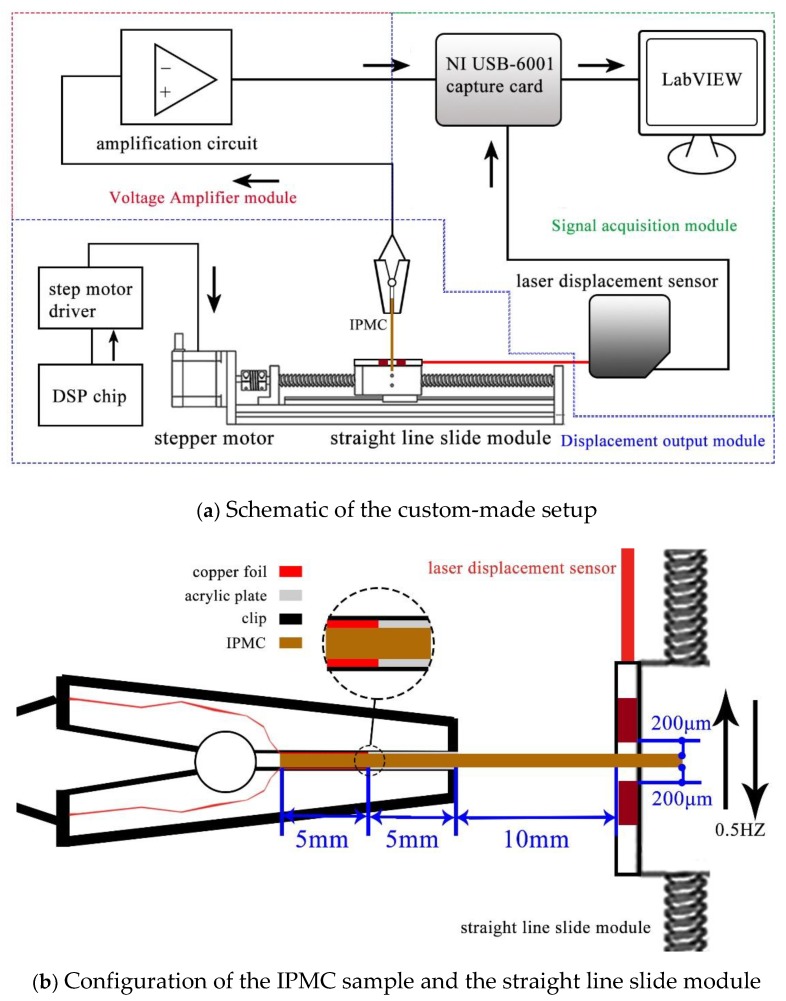
Sensing test platform for the IPMC sample.

The sensing behavior of IPMC was realized using a self-made measuring system, which consisted of an excitation, record, and signal amplifier module. When the IPMC sample was applied with an ideal stimulus signal, generated by the excitation module, the signal amplifier module perceived the weak electrical signal, amplified it over 100 times, and the record module was responsible for transferring and recording data. The total device flow chart is shown in the Figure 5a.

As shown in Figure 5b, the deformation generated by the stepping motor was transformed into a reciprocating displacement signal through the lead screw, which was applied to one end of IPMC sample. Due to leaving a small gap, the clamp was not fixed to the IPMC sample. The straight line slide module moved at a speed of 2 mm/s, with a period of 2 s. The other end of the IPMC sample was clamped with a clip fixed on the laboratory bench, and formed a cantilever structure. The part of the clip that was in contact with the IPMC was divided into two sections. The left half was affixed with copper foil, which can send the signal generated by IPMC to the amplification module. The right half of the fixed acrylic sheet could be used to further fix the IPMC to prevent the thicker (greater stiffness coefficient) IPMC from prying up the clip during the bending process, resulting in the capacitance to interfere with the experimental results. The free length between the retaining clip and the point of application for displacement was set to 10 mm. 

The signal generated by the IPMC sample was transmitted to the custom-made circuit through the copper electrodes inside the clip and then sent to the acquisition board. There were two kinds of amplification circuits, which amplified the voltage and the current signals, respectively. The signal generated by the displacement output module was measured by a Keyence IL-065 laser displacement sensor. All data were recorded using a National Instruments USB-6001 data acquisition board at a sampling rate of 1000/s.

## 3. Results and Discussion

In general, we tested the IPMC sample in an open environment. During the test, the water molecules inside the IPMC will gradually evaporate and the water content of the IPMC decreases, which will affect the sensing signal. In order to eliminate this effect, we measured the sample after it was taken from DI water for 10 min. 

### 3.1. Comparison of the Current Amplification and the Voltage Amplification

**Figure 6:**
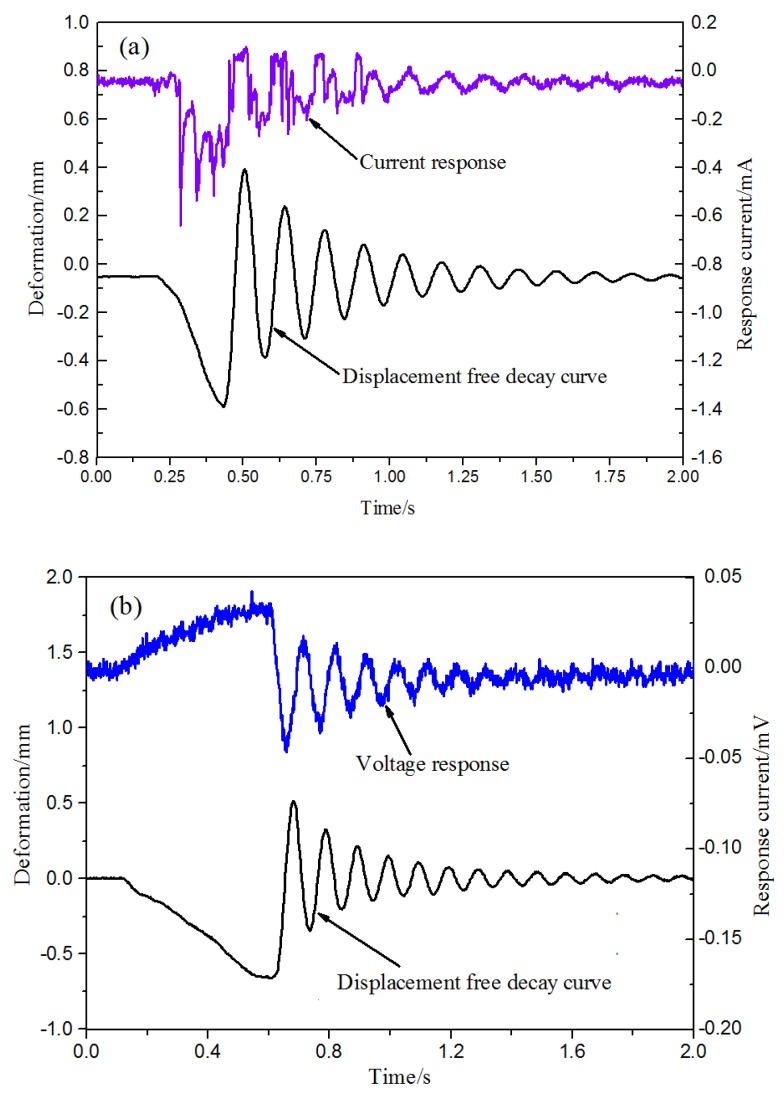
Comparison of the current amplification and the voltage amplification: (**a**) amplify the current of the IPMC sensor signal and (**b**) Amplify the voltage of the IPMC sensor signal.

Figure 6 shows the signal generated by the L3 sample under free oscillation through the current and voltage amplification circuits, respectively. It can be seen that the sensing signal curve in Figure 6b is significantly closer to the displacement curve. Compared with the current response, the voltage response more intuitively reflects the distribution of the hydrated cations, which helps to reveal the sensing physics. In the following tests, we selected the voltage amplification circuit to amplify the IPMC sensing signal.

**Figure 7:**
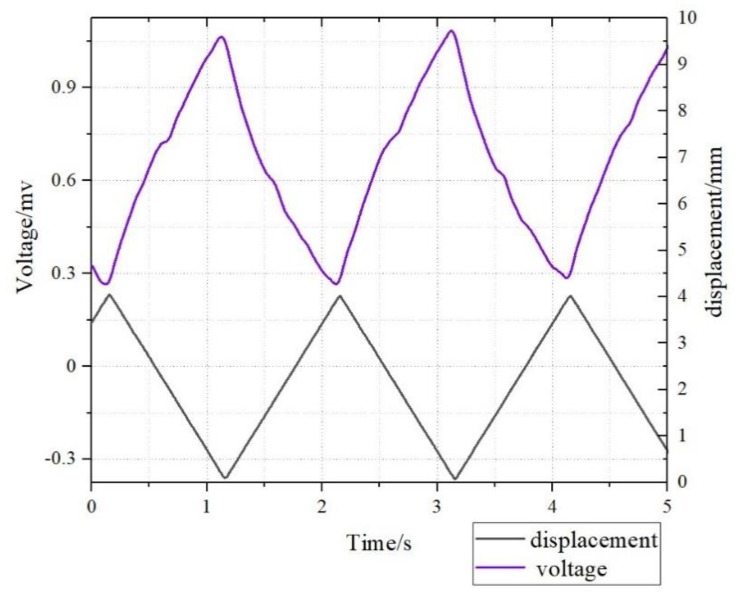
The deformation sensing of the L3 sample with triangular wave signal.

The free oscillations in Figure 6 were replaced with the displacement output module, and the voltage signal generated by the L3 sample as shown in Figure 7. When the dynamic deformation of 2 mm/s and 0.5 Hz was applied, the maximum voltage generated by the L3 sample increased to 0.9 mV.

### 3.2. Thickness Effect on the Deformation Sensing of IPMC

**Figure 8:**
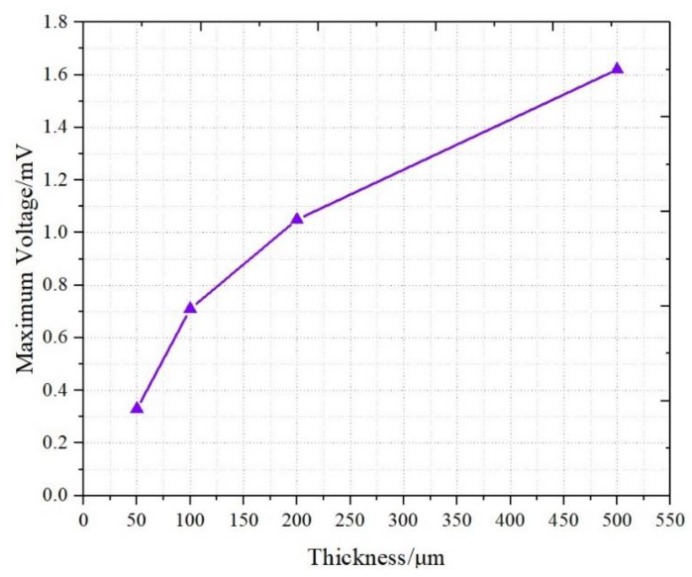
Signals of different thicknesses.

Figure 8 demonstrates the relationship between the maximum response voltage and the thickness of the IPMC. The results revealed that the voltage response increased with an increase in the thickness of the sample. The 50 μm sample (T1) produced the smallest voltage signal at 0.32 mV and the 500 μm sample (T4) produced the largest voltage signal at 1.61 mV. In addition to the stress distribution, we also tried to use the distribution of cations on the inner surface to explain the increase response voltage in response to thickness.

**Figure 9:**
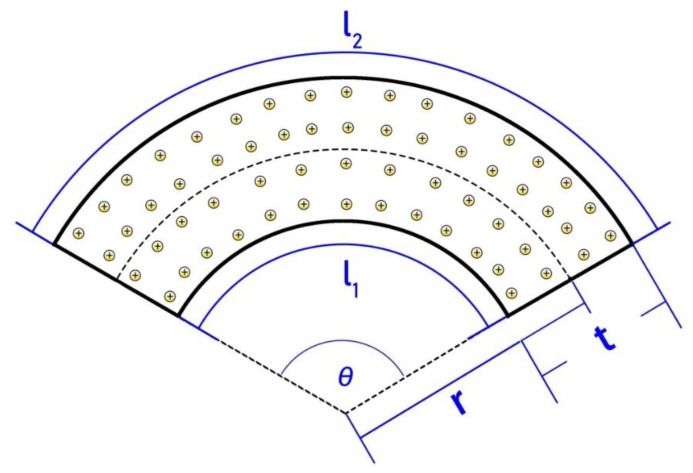
IPMC in the bending state.

As mentioned in Figure 1, when applying a bending deformation, an elastic stress gradient is generated along the thickness, thus the mobile cations and water molecules migrate towards the outside electrode. After ionic charges redistribute along the thickness, cations were also redistributed near the two inner surfaces of the electrode. As shown in Figure 9, after deformation, the two lengths of the IPMC surface are *l_1_* and *l_2_*, respectively. The sizes (*w*, *l,* and *t*) and the difference (δ) of cation numbers on the two internal surfaces of the IPMC inevitably presented a proportional relationship, which could be qualitatively depicted by the following Equation (1):

(1) $$\delta =N_{2}-N_{1}=\left(S_{2}-S_{1}\right)\rho =wt\theta \rho ,$$

where *N_1_* and *N_2_* are the number of cations on the internal surface of IPMC, respectively, *S*_1_ and *S*_2_ are the surface areas of IPMC, *r* is the bending radius of IPMC, *w, t,* and *l* are the width, thickness, and length of IPMC sample, respectively, and *ρ* is the density of cations on the inner surface. As can be seen in (1), the difference (δ) is positively correlated with the thickness (*t*) of IPMC.

Aureli et al. found that the capacitance of IPMC was largely dictated by the effective electrode surface area rather than the thickness, when the Debye screening length was considerably smaller than the polymer thickness [24]. When the surface electrode morphology of IPMC samples are similar, the capacitance (*C*) is positively correlated with the surface areas(*S*) of IPMC, as shown in Equation (2):

(2) C∝S=wl.

Then the voltage response of IPMC and the dimensions present a proportional relationship, which could be qualitatively depicted by the following Equation (3):

(3) $$U=\frac{\delta }{C}\propto \frac{t}{l}\theta \rho .$$

As can be seen in (3), the voltage response of the IPMC (*U*) is positively correlated with the thickness (*t*) of IPMC and negatively correlated with length.

### 3.3. The Effect of Length on Deformation Sensing of IPMC

**Figure 10:**
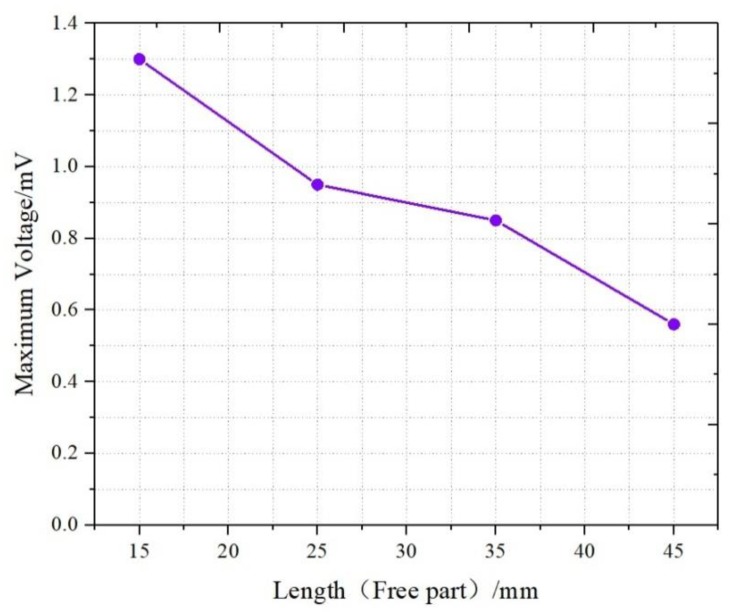
Signals of different lengths (Free part).

Figure 10 shows the relationship between the maximum voltage and the length of the IPMC. The length between the retaining clip and the point of application for displacement was set to 10 mm. As the length increased, the voltage signal decreased, and the voltage generated by the L4 sample was only 0.56 mV. This trend was consistent with Equation (3). With a length increase in the IPMC, the capacitance increased accordingly. When the same bending displacement was applied and the generated charges were equal, the electrical response was dominated by the length of the IPMC sample.

### 3.4. The Effect of Width on Deformation Sensing of IPMC

**Figure 11:**
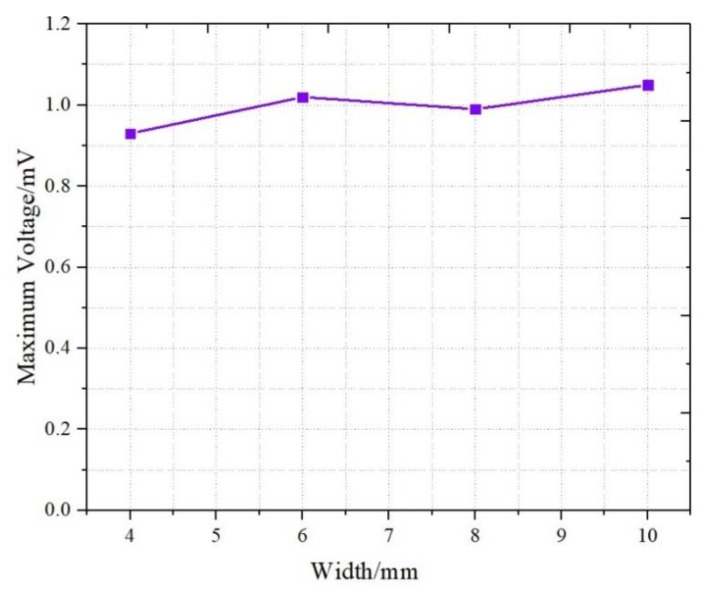
Signals of different widths.

The relationship between the maximum voltage and the width of the IPMC is displayed in Figure 11. The results showed that the voltage does not change significantly as the width increased. The voltage of the four samples of W1–W4 was basically distributed around 1 mV. According to Equation (2), an increase in the IPMC width resulted in the increase of the capacitance. Meanwhile, as shown in Equation (1), the wider IPMC had a larger deformation area when the same displacement was applied, and then the difference (δ) of cation numbers near the two internal surfaces of the IPMC increased correspondingly. Taking the two factors into consideration (see Equation (3)), the effect of IPMC width was offset, and the response trend of the IPMC in width was not so obvious. From the experimental results, the signal response of IPMC with various dimensions could be depicted by the Equation (3). Table 2 summarizes the results of the maximum response signals of the IPMC samples.

## 4. Conclusions

In this study, the Pd-Au typed IPMCs with various thicknesses, widths, and lengths were fabricated and their sensing performances were tested using a self-made electromechanical sensing test platform. Current amplification and voltage amplification were performed on the signals generated during the IPMC bending. By comparison, it was found that the response signal was closer to the displacement curve after passing through the voltage amplification module. The minimum voltage signal (0.32 mV) was generated by the sample of T1 and the maximum voltage signal (1.61 mV) was generated by the sample of T4. A thicker IPMC achieved better sensor performance and, in contrast, a longer IPMC, when subjected to equal bending, the response voltage decreased. The change in width has a weaker effect on the sensing signal. A simplified static model was employed to explain the sensing properties of IPMC with various sizes. In order to obtain stronger sensing signals under the same bending, the thickness of the Nafion membrane can be appropriately increased or its length can be shortened.

## Figures and Tables

**Table 1:** The size parameters of the ionic polymer-metal composite (IPMC).

No.	Thickness (μm)	Length (mm)	Clamped Part (mm)	Free Part (mm)	Width (mm)
T1	50	30	10	20	5
T2	100	30	10	20	5
T3	200	30	10	20	5
T4	500	30	10	20	5
L1	200	25	10	15	5
L2	200	35	10	25	5
L3	200	45	10	35	5
L4	200	55	10	45	5
W1	200	30	10	20	4
W2	200	30	10	20	6
W3	200	30	10	20	8
W4	200	30	10	20	10

**Table 2:** Response signals of the IPMC samples.

Samples	T1	T2	T3	T4	L1	L2	L3	L4	W1	W2	W3	W4
Response Signals (mV)	0.33	0.71	1.05	1.62	1.3	0.95	0.85	0.56	0.93	1.02	0.99	1.05
